# Supplementary material for: Assessing the Feasibility and Efficacy of Pre-Sleep Dim Light Therapy for Adults with Insomnia: A Pilot Study
Source: Medicina (Kaunas). 2024 Apr 14;60(4):632. doi: 10.3390/medicina60040632 (PMC11052339; doi:10.3390/medicina60040632)
Supplement: Supplementary file 1 [file medicina-60-00632-s001.zip › medicina-2939603-supplementary.pdf]

Supplementary Materials

R codes used in the statistical analysis

```
library(pwr)
library(openxlsx)
library(dplyr)
library(tidyr)
library(gtsummary)
library(ggplot2)
library(ggbeeswarm)
library(patchwork)
library(lmerTest)
library(multcomp)
library(survminer)
library(stringr)
library(rstatix)
library(signs)
library(rms)

select <- dplyr::select
theme_gtsummary_compact()
mean_sd <- function(x) {paste0(sprintf('%.2f',mean(x,na.rm=T)),' ± ',sprintf('%.2f',sd(x,na.rm=T)))}

# Table 1 ------------------------------------------------------------------------------------

data %>%
  mutate(Gender = factor(Gender, levels=c('F','M'), labels=c('Women','Men')),
         BMI = Wt/(Ht/100)^2,
         DM = factor(DM, levels=c(1,0), labels=c('Yes','No')),
         HTN = factor(HTN, levels=c(1,0), labels=c('Yes','No')),
         dyslipidemia = factor(if_else(dyslipidemia==1, 1, 0), levels=c(1,0), labels=c('Yes','No')),
         Smoke = factor(if_else(Smoke1==0, 0, 1), levels=c(1,0), labels=c('Yes','No')),
         Drinking = factor(if_else(Drinking1==0, 0, 1), levels=c(1,0), labels=c('Yes','No')),
         Exercise = factor(if_else(`Exercise_1-1`==0&`Exercise_2-1`==0, 0, 1), levels=c(1,0), labels=c('Yes','No')),
         Smoke2 = factor(if_else(Smoke2==0, 0, 1), levels=c(1,0), labels=c('Yes','No')),
         Drinking2 = factor(if_else(Drinking2==0, 0, 1), levels=c(1,0), labels=c('Yes','No')),
         Exercise2 = factor(if_else(`Exercise_1-2`==0&`Exercise_2-2`==0, 0, 1), levels=c(1,0),
labels=c('Yes','No'))) %>%
  select(group, age, Gender, DM, HTN, dyslipidemia, SBP, DBP, Wt, Ht, BMI, Smoke, Drinking, Exercise, GSDS) %>%
  tbl_summary(by = group,
              type = list(where(is.numeric) ~ 'continuous',
                          where(is.factor) ~ 'categorical'),
              statistic = list(all_continuous() ~ '{mean} ± {sd}',
                               all_categorical() ~ '{n} ({p}%)'),
              digits = list(all_continuous() ~ 2,
                            all_categorical() ~ c(0, 1))) %>%
  add_p(test = list(all_categorical() ~ 'fisher.test',
                    all_continuous() ~ 't.test'),
        pvalue_fun = function(x) ifelse(x<0.001, '<0.001', sprintf('%.3f', x))) %>%
  add_overall()

# Table 2 ------------------------------------------------------------------------------------

tab2_df <- data %>%
  mutate(group = if_else(group=='A', 1, 0)) %>%
  select(group, age, Gender, BMI, HTN, DM, dyslipidemia, WBC_1:Serotonin_2) %>%
  rename_with(~ sub('\\_1|\\-1', '_pre', .)) %>% rename_with(~ sub('\\_2|\\-2', '_post', .)) %>%
  mutate_at(vars(WBC_pre:Serotonin_post), extract_numeric)

u_var <- c('WBC','Glucose','Insulin','HOMA.IR.index','Cholesterol.Total','Triglyceride',
           'HDL','LDL','CRP','Cortisol','ACTH','Serotonin')
res <- list()
for(i in 1:length(u_var)){
  sub_df <- tab2_df %>%
```

```
        select(group, age, Gender, BMI, HTN, DM, dyslipidemia, contains(u_var[i])) %>%
        rename_with(~ sub(paste0(u_var[i],'_'), '', . ,fixed=T),
                    contains(u_var[i])) %>%
        mutate(diff = post - pre)

    tmp <- sub_df %>%
        mutate(group = ifelse(group==1, 'A', 'B')) %>%
        group_by(group) %>%
        summarise(Pre = mean_sd(pre),
                  Post = mean_sd(post),
                  Diff = mean_sd(diff),
                  p = t.test(diff)$p.value) %>%
        mutate(p = ifelse(p<0.001, '<0.001', sprintf('%.3f',p))) %>%
        pivot_wider(names_from=group,
                    values_from=Pre:p,
                    names_vary='slowest')

    lm_fit <- lm(diff ~ pre + group + age + Gender + BMI + HTN + DM + dyslipidemia, sub_df)
    did <- sprintf('%.2f',summary(lm_fit)$coefficient[3,1])
    lc <- sprintf('%.2f',confint(lm_fit, parm='group')[1])
    uc <- sprintf('%.2f',confint(lm_fit, parm='group')[2])
    p <- summary(lm_fit)$coefficient[3,4]
    p <- ifelse(p<0.001, '<0.001', sprintf('%.3f',p))

    res[[i]] <- cbind(variable=u_var[i], tmp, DID=paste0(did,' (',lc,', ',uc,')'), p=p)}

do.call('rbind', res)

# Table 3 ---------------------------------------------------------------------------------

tab3_df <- data %>%
    mutate(group = if_else(group=='A', 1, 0),
           Gender = if_else(Gender=='M', 1, 0)) %>%
    select(No, group, age, Gender, BMI, DM, HTN, dyslipidemia, sleep.efficiency_1:Avg.Awakening_avg) %>%
    select(-contains('_avg')) %>%
    pivot_longer(cols=sleep.efficiency_1:Avg.Awakening_14,
                 names_sep = '_',
                 names_to = c('var','time')) %>%
    mutate(time = as.numeric(time))

u_var <- unique(tab3_df$var)
res <- p_dat <- list()
for(i in 1:length(u_var)){

    stat <- tab3_df %>%
        filter(var==u_var[i]) %>%
        mutate(group = if_else(group==1, 'A', 'B')) %>%
        group_by(group) %>%
        summarise(value = mean_sd(value)) %>%
        pivot_wider(names_from=group,
                    values_from=value)

    lmm_fit <- lmer(value ~ group + BMI + (1|No), tab3_df %>% filter(var==u_var[i]))
    # lmm_fit <- lmer(value ~ group + BMI + DM + HTN + dyslipidemia + (1|No), tab3_df %>% filter(var==u_var[i]))

    diff <- sprintf('%.2f',summary(lmm_fit)$coefficient[2,1])
    lc <- sprintf('%.2f',confint.merMod(lmm_fit, parm='group')[1])
    uc <- sprintf('%.2f',confint.merMod(lmm_fit, parm='group')[2])
    p <- summary(lmm_fit)$coefficient[2,5]
    p <- ifelse(p<0.001, '<0.001', sprintf('%.3f',p))

    p_dat[[i]] <- tab3_df %>%
        filter(var==u_var[i]) %>%
        mutate(group = if_else(group==1, 'A', 'B')) %>%
        group_by(group) %>%
        summarise(mean = mean(value),
```

```
                sd = sd(value))
  res[[i]] <- cbind(stat, Diff=paste0(diff,' (',lc,', ',uc,')'), p=p)
}

do.call('rbind', res)

# Figure 2 ---------------------------------------------------------------

tab_df <- data %>%
  mutate(BMI = Wt/(Ht/100)^2) %>%
  select(group, age, Gender, BMI, DM, HTN, dyslipidemia, 일주기_PRE, 일주기_POST, ESS_PRE, ESS_POST,
         ISI_PRE, ISI_POST, PHQ_PRE, PHQ_POST, PSQI_PRE, PSQI_POST, SSS_PRE, SSS_POST) %>%
  rename_with(~ sub('\\_POST', '_post', .)) %>% rename_with(~ sub('\\_PRE', '_pre', .)) %>%
  mutate(group = if_else(group=='A', 1, 0),
         Gender = if_else(Gender=='M', 1, 0)) %>%
  mutate_all(as.numeric)

long_df <- tab_df %>%
  mutate(across(ends_with('_post'), .names = 'diff_{col}') - across(ends_with('_pre'))) %>%
  pivot_longer(cols = 일주기_pre:SSS_post,
               names_to = c('variable', 'time'),
               names_sep = '\\_')

bar_df <- long_df %>%
  group_by(variable, group, time) %>%
  summarise(mean = mean(value, na.rm=T), sd = sd(value, na.rm=T))

u_var <- c('일주기','ESS','ISI','PHQ','PSQI','SSS')
titles <- c('MEQ','ESS','ISI','PHQ-9','PSQI','SSS')

p <- list()
for(i in 1:length(u_var)){

  bar_df_sub <- bar_df %>%
    mutate(time = factor(if_else(time=='pre', 'Pre', 'Post'), levels=c('Pre','Post')),
           group = factor(group, levels=c(0,1), labels=c('Control','LT'))) %>%
    filter(variable==u_var[i])
  m <- max(bar_df_sub$mean+bar_df_sub$sd)

  bar_p <- bar_df_sub %>%
    ggplot(aes(x=group, y=mean)) +
    geom_bar(aes(group=time, fill=time), stat='identity', color='black', position=position_dodge(), width=0.85) +
    geom_errorbar(aes(ymin=mean-sd, ymax=mean+sd, group=time), width=.2, position=position_dodge(.9)) +
    labs(y='Mean Scores') +
    coord_cartesian(ylim = c(0, m*1.2), clip = 'off') +
    annotate("text", c(0.8, 1.2, 1.8, 2.2), y = 0, label = c('Pre','Post','Pre','Post'), vjust=2.25, size=3.5) +
    annotate("text", c(1,2), y = 0, label = c('Control','LT'), vjust=4, size=3.5) +
    scale_y_continuous(limits=c(-2.5, m*1.2)) +
    theme_bw() +
    theme(
      plot.margin = unit(c(1, 1, 2.5, 1), 'lines'),
      legend.position = 'none',
      axis.title.x = element_blank(),
      axis.text.x = element_blank(),
      axis.text.y = element_text(size=10, color='black'),
      axis.ticks.x = element_blank(),
      axis.title.y = element_text(size=10)
    )

  sub_df <- tab_df %>%
    select(group, age, Gender, BMI, DM, HTN, dyslipidemia, contains(u_var[i])) %>%
    rename_with(~ sub(paste0(u_var[i],'_'), '', . ,fixed=T),
                contains(u_var[i])) %>%
    mutate(diff = post - pre)
```

```
tmp <- sub_df %>%
  group_by(group) %>%
  summarise(Pre = mean_sd(pre),
            Post = mean_sd(post),
            Diff = mean_sd(diff),
            p = t.test(diff)$p.value,
            p = sprintf('%.3f',p)) %>%
  pivot_wider(names_from=group,
              values_from=Pre:p,
              names_vary='slowest')

fit <- lm(diff~group, sub_df)
diff_ci <- sprintf('%.3f',as.vector(confint(fit, parm='group')))
unadj <- data.frame(variable=u_var[i], type='unadj', diff=as.vector(fit[[1]][2]),
                    lower=diff_ci[1], upper=diff_ci[2])

fit <- lm(diff ~ pre + group + BMI + DM + HTN + dyslipidemia, sub_df)
# fit <- lm(diff ~ pre + group + BMI, sub_df)
diff_ci <- sprintf('%.3f',as.vector(confint(fit, parm='group')))
adj <- data.frame(variable=u_var[i], type='adj', diff=as.vector(fit[[1]][3]),
                  lower=diff_ci[1], upper=diff_ci[2])

diff_list <- rbind(unadj, adj)

if(tmp$p_0<0.05){
  m1 <- max(bar_df_sub$mean[bar_df_sub$group=='Control']+
            bar_df_sub$sd[bar_df_sub$group=='Control'])
  sig_df <- data.frame(group = c(0.8,0.8,1.2,1.2),
                       mean = m1 * c(1.05, 1.1, 1.1, 1.05))
  bar_p <- bar_p +
    geom_line(aes(x=group, y=mean), sig_df) +
    annotate('text', x = 1, y = m1*1.15, label = '*', size = 5)
}

if(tmp$p_1<0.05){
  m2 <- max(bar_df_sub$mean[bar_df_sub$group=='LT']+
            bar_df_sub$sd[bar_df_sub$group=='LT'])
  sig_df <- data.frame(group = c(1.8,1.8,2.2,2.2),
                       mean = m2 * c(1.05, 1.1, 1.1, 1.05))
  bar_p <- bar_p +
    geom_line(aes(x=group, y=mean), sig_df) +
    annotate('text', x = 2, y = m2*1.15, label = '*', size = 5)
}

diff_df_sub <- diff_list %>%
  filter(type=='adj') %>%
  mutate(type = factor(type, levels=c('adj'), labels=c('Adjusted')),
         lower = as.numeric(lower),
         upper = as.numeric(upper))

diff_p <- diff_df_sub %>%
  ggplot() +
  geom_pointrange(aes(x=type, y=diff, ymin=lower, ymax=upper)) +
  geom_hline(yintercept=0, linetype=2, linewidth=0.5) +
  labs(y='Difference in Difference') +
  scale_y_continuous(limits=c(min(diff_df_sub$lower)-2, max(diff_df_sub$upper)+2), labels=signs_format(accuracy
= 1)) +
  theme_bw() +
  theme(
    axis.title.x = element_blank(),
    axis.title.y = element_text(size=10),
    axis.text = element_text(size=10, color='black')
  )

sig_vec <- diff_df_sub$lower*diff_df_sub$upper>0
```

```
  if(any(sig_vec)){
    sig_df <- data.frame(type='Adjusted', y=diff_df_sub$upper[sig_vec])
    diff_p <- diff_p + geom_point(aes(y=y+1, x=type), sig_df, shape=42, size=5)
  }

  title <- grid::textGrob(label=titles[i], gp=grid::gpar(cex=0.9))
  p[[i]] <- (wrap_elements(panel = title) / (bar_p + diff_p + plot_layout(widths=c(0.7,0.3), tag_level = 'new'))) +
    plot_layout(height=c(0.09,0.91))}

wrap_plots(p, ncol=3) + plot_annotation(tag_levels='A')

# Figure 3 ---------------------------------------------------------------

saliva <- data %>%
  select(No, group, contains('Melatonin')) %>%
  rename(ID = No) %>%
  pivot_longer(cols = `Melatonin._1-1am`:`Melatonin._2-10pm`,
               names_to = c('variable', 'time'),
               names_sep = c('\\._')) %>%
  mutate(g = factor(group),
         seq = factor(ifelse(substr(time,1,1)%in%c('1'), 'Before', 'After'), levels=c('Before','After')),
         group = factor(group, levels=c('B','A'), labels=c('Control','LT')),
         ID_seq = paste(ID, substr(time,1,1), sep='_'),
         time = as.numeric(substr(time,3,3)),
         value = case_when(value=='>50.000' ~ 50,
                           value=='<0.780' ~ 0.78,
                           TRUE ~ as.numeric(value))) %>%
  group_by(ID, seq) %>%
  arrange(value, .by_group=T) %>%
  mutate(time=1:n())

fit1 <- lm(value~rcs(time,3), saliva %>% filter(group=='LT', seq=='Before'))
fit2 <- lm(value~rcs(time,3), saliva %>% filter(group=='LT', seq=='After'))

time <- seq(1,10,by=0.01)
LT_before <- predict(fit1, newdata = data.frame(time=time))
LT_after <- predict(fit2, newdata = data.frame(time=time))

t <- seq(1,10,by=0.0001)
value_before <- predict(fit1, newdata = data.frame(time=t))
value_after <- predict(fit2, newdata = data.frame(time=t))

x1 <- t[which.min(abs(value_before-12.667))]
x2 <- t[which.min(abs(value_after-13.855))]
y1 <- value_before[which.min(abs(value_before-12.667))]
y2 <- value_after[which.min(abs(value_after-13.855))]

p1 <- data.frame(time=c(time,time),
                 value=c(LT_before,LT_after),
                 seq=rep(c('Before','After'),each=length(time)) %>%
                   factor(levels=c('Before','After'))) %>%
  ggplot(aes(x=time, y=value)) +
  geom_smooth(aes(color=seq), size=1.3, se=F, method=lm, formula=y~rcs(x,3)) +
  scale_color_manual(values = c('#F5A94D','#7865A5')) +
  geom_segment(aes(x=x1, y=0, yend=y1, xend=x1), linewidth=0.6) +
  geom_segment(aes(x=1, y=y1, yend=y1, xend=x1), linewidth=0.6) +
  geom_segment(aes(x=x2, y=0, yend=y2, xend=x2), linewidth=0.6) +
  geom_segment(aes(x=1, y=y2, yend=y2, xend=x2), linewidth=0.6) +
  geom_point(data=data.frame(x=c(x1,x2),y=c(y1,y2)), aes(x=x,y=y), size=2) +
  scale_x_continuous(breaks=c(1:10), expand=c(0,0)) +
  scale_y_continuous(limits=c(0,40), expand=c(0,0)) +
  labs(x='Time', y='Melatonin', title='LT') +
  theme_classic2() +
  theme(plot.title = element_text(size=13, hjust=0.5, face='bold'),
        strip.background = element_blank(),
        legend.title = element_blank(),
```

```
            axis.title = element_text(size=11, color='black'),
            axis.text = element_text(size=11, color='black'),
            axis.text.x = element_text(size=11),
            legend.position = 'bottom',
            legend.text = element_text(size=11))

fit1 <- lm(value~rcs(time,3), saliva %>% filter(group=='Control', seq=='Before'))
fit2 <- lm(value~rcs(time,3), saliva %>% filter(group=='Control', seq=='After'))

time <- seq(1,10,by=0.01)
LT_before <- predict(fit1, newdata = data.frame(time=time))
LT_after <- predict(fit2, newdata = data.frame(time=time))

t <- seq(1,10,by=0.0001)
value_before <- predict(fit1, newdata = data.frame(time=t))
value_after <- predict(fit2, newdata = data.frame(time=t))

a1 <- t[which.min(abs(value_before-17.511))]
a2 <- t[which.min(abs(value_after-18.551))]
b1 <- value_before[which.min(abs(value_before-17.511))]
b2 <- value_after[which.min(abs(value_after-18.551))]

p2 <- data.frame(time=c(time,time),
                 value=c(LT_before,LT_after),
                 seq=rep(c('Before','After'),each=length(time)) %>%
                   factor(levels=c('Before','After'))) %>%
  ggplot(aes(x=time, y=value)) +
  geom_smooth(aes(color=seq), size=1.3, se=F, method=lm, formula=y~rcs(x,3)) +
  scale_color_manual(values = c('#F5A94D','#7865A5')) +
  geom_segment(aes(x=a1, y=0, yend=b1, xend=a1), linewidth=0.6) +
  geom_segment(aes(x=1, y=b1, yend=b1, xend=a1), linewidth=0.6) +
  geom_segment(aes(x=a2, y=0, yend=b2, xend=a2), linewidth=0.6) +
  geom_segment(aes(x=1, y=b2, yend=b2, xend=a2), linewidth=0.6) +
  geom_point(data=data.frame(x=c(a1,a2),y=c(b1,b2)), aes(x=x,y=y), size=2) +
  scale_x_continuous(breaks=c(1:10), expand=c(0,0)) +
  scale_y_continuous(limits=c(0,40), expand=c(0,0)) +
  labs(x='Time', y='Melatonin', title='Control') +
  theme_classic2() +
  theme(plot.title = element_text(size=13, hjust=0.5, face='bold'),
            strip.background = element_blank(),
            legend.title = element_blank(),
            axis.title = element_text(size=11, color='black'),
            axis.text = element_text(size=11, color='black'),
            axis.text.x = element_text(size=11),
            legend.position = 'bottom',
            legend.text = element_text(size=11))

p2 + p1 + plot_annotation(tag_levels = 'A') + plot_layout(guides='collect') & theme(legend.position = 'bottom')

# Figure 4 ---------------------------------------------------------------

tab_df <- data %>%
  mutate(group = if_else(Group=='실험군', 1, 0)) %>%
  select(group, CLOCK_1:`REV-ERBb_2`) %>%
  rename_with(~ sub('\\_1|\\-1', '_pre', .)) %>% rename_with(~ sub('\\_2|\\-2', '_post', .)) %>%
  mutate_at(vars(CLOCK_pre:`REV-ERBb_post`), as.numeric)

u_var <- unique(gsub('\\_pre|_post','',names(tab_df)[-c(1)]))
res <- list(); diff_list <- list()
for(i in 1:length(u_var)){
  sub_df <- tab_df %>%
    select(group, contains(u_var[i])) %>%
    rename_with(~ sub(paste0(u_var[i],'_'), '', . ,fixed=T),
                contains(u_var[i])) %>%
    mutate(diff = post - pre)
```

```
  tmp <- sub_df %>%
    group_by(group) %>%
    summarise(Pre = mean_sd(pre),
              Post = mean_sd(post),
              Diff = mean_sd(diff),
              p = t.test(diff)$p.value,
              p = sprintf('%.3f',p)) %>%
    pivot_wider(names_from=group,
                values_from=Pre:p,
                names_vary='slowest')

  fit <- lm(diff~group, sub_df)
  diff_ci <- sprintf('%.3f',as.vector(confint(fit, parm='group')))
  unadj <- data.frame(variable=u_var[i], type='unadj', diff=as.vector(fit[[1]][2]),
                      lower=diff_ci[1], upper=diff_ci[2])

  fit <- lm(diff ~ pre + group, sub_df)
  diff_ci <- sprintf('%.3f',as.vector(confint(fit, parm='group')))
  adj <- data.frame(variable=u_var[i], type='adj', diff=as.vector(fit[[1]][3]),
                    lower=diff_ci[1], upper=diff_ci[2])

  did <- sprintf('%.2f',summary(fit)$coefficient[3,1])
  lc <- sprintf('%.2f',confint(fit, parm='group')[1])
  uc <- sprintf('%.2f',confint(fit, parm='group')[2])
  p <- summary(fit)$coefficient[3,4]
  p <- ifelse(p<0.001, '<0.001', sprintf('%.3f',p))

  res[[i]] <- cbind(variable=u_var[i], tmp, DID=paste0(did,' (',lc,', ',uc,')'), p=p)
  diff_list[[i]] <- rbind(unadj, adj)
}

do.call('rbind', res)

long_df <- tab_df %>%
  mutate(across(ends_with('_post'), .names = 'diff_{col}') - across(ends_with('_pre'))) %>%
  pivot_longer(cols = CLOCK_pre:`REV-ERBb_post`,
               names_to = c('variable', 'time'),
               names_sep = '\\_')

bar_df <- long_df %>%
  group_by(variable, group, time) %>%
  summarise(mean = mean(value, na.rm=T), sd = sd(value, na.rm=T))

diff_df <- do.call('rbind', diff_list)

titles <- c('CLOCK','BMAL1','PER1','PER2','PER3','CRY1','CRY2','REV-ERBa','REV-ERBb')
p <- list()
for(i in 1:length(u_var)){

  bar_df_sub <- bar_df %>%
    mutate(time = factor(if_else(time=='pre', 'Pre', 'Post'), levels=c('Pre','Post')),
           group = factor(group, levels=c(0,1), labels=c('Control','LT'))) %>%
    filter(variable==u_var[i])
  m <- max(bar_df_sub$mean+bar_df_sub$sd)

  bar_p <- bar_df_sub %>%
    ggplot(aes(x=group, y=mean)) +
    geom_bar(aes(group=time, fill=time), stat='identity', color='black', position=position_dodge(), width=0.85) +
    geom_errorbar(aes(ymin=mean-sd, ymax=mean+sd, group=time), width=.2, position=position_dodge(.9)) +
    labs(y='Mean Scores') +
    coord_cartesian(ylim = c(0, m*1.2), clip = 'off') +
    annotate("text", c(0.8, 1.2, 1.8, 2.2), y = 0, label = c('Pre','Post','Pre','Post'), vjust=2.25, size=3.5) +
    annotate("text", c(1,2), y = 0, label = c('Control','LT'), vjust=4, size=3.5) +
    scale_y_continuous(limits=c(-2.5, m*1.2)) +
    theme_bw() +
```

```
    theme(
      plot.margin = unit(c(1, 1, 2.5, 1), 'lines'),
      legend.position = 'none',
      axis.title.x = element_blank(),
      axis.text.x = element_blank(),
      axis.text.y = element_text(size=10, color='black'),
      axis.ticks.x = element_blank(),
      axis.title.y = element_text(size=10)
    )

  if(res[[i]]$p_0<0.05){
    m1 <- max(bar_df_sub$mean[bar_df_sub$group=='Control']+
              bar_df_sub$sd[bar_df_sub$group=='Control'])
    sig_df <- data.frame(group = c(0.8,0.8,1.2,1.2),
                         mean = m1 * c(1.05, 1.1, 1.1, 1.05))
    bar_p <- bar_p +
      geom_line(aes(x=group, y=mean), sig_df) +
      annotate('text', x = 1, y = m1*1.15, label = '*', size = 5)
  }

  if(res[[i]]$p_1<0.05){
    m2 <- max(bar_df_sub$mean[bar_df_sub$group=='LT']+
              bar_df_sub$sd[bar_df_sub$group=='LT'])
    sig_df <- data.frame(group = c(1.8,1.8,2.2,2.2),
                         mean = m2 * c(1.05, 1.1, 1.1, 1.05))
    bar_p <- bar_p +
      geom_line(aes(x=group, y=mean), sig_df) +
      annotate('text', x = 2, y = m2*1.15, label = '*', size = 5)
  }

  diff_df_sub <- diff_df %>%
    filter(type=='adj') %>%
    mutate(type = factor(type, levels=c('adj'), labels=c('Adjusted')),
           lower = as.numeric(lower),
           upper = as.numeric(upper)) %>%
    filter(variable==u_var[i])

  diff_p <- diff_df_sub %>%
    ggplot() +
    geom_pointrange(aes(x=type, y=diff, ymin=lower, ymax=upper)) +
    geom_hline(yintercept=0, linetype=2, size=0.5) +
    labs(y='Difference in Difference') +
    scale_y_continuous(limits=c(min(diff_df_sub$lower)-2, max(diff_df_sub$upper)+2), labels=signs_format(accuracy
= 1)) +
    theme_bw() +
    theme(
      axis.title.x = element_blank(),
      axis.title.y = element_text(size=10),
      axis.text = element_text(size=10, color='black')
    )

  sig_vec <- diff_df_sub$lower*diff_df_sub$upper>0
  if(any(sig_vec)){
    sig_df <- data.frame(type='Adjusted', y=diff_df_sub$upper[sig_vec])
    diff_p <- diff_p + geom_point(aes(y=y+1, x=type), sig_df, shape=42, size=5)
  }

  title <- grid::textGrob(label=titles[i], gp=grid::gpar(cex=0.9))
  p[[i]] <- (wrap_elements(panel = title) / (bar_p + diff_p + plot_layout(widths=c(0.7,0.3), tag_level = 'new'))) +
    plot_layout(height=c(0.09,0.91))

}

wrap_plots(p, ncol=3) + plot_annotation(tag_levels='A')
```

EVERFINE

Spectrum Test Report

Sample	:	Date	: 2023-11-08 13:07:25
Specification	:	Sam. Status	:
Sample No.	: 1	Standard	:
Manufacturer	:	Instrument	: HaasSuite(EVERFINE)
Assessor	: damin		
Remark	:	Test by	:
Device SN	:		

Test Condition

Temprature	: 24.5Deg	RH	: 48%
WL Range	: 380nm-780nm	IP	: 46383 (71%)
Test Mode	: Accuracy Test	T	: 7746 ms
Sensitivity	: Low		

Spectrum

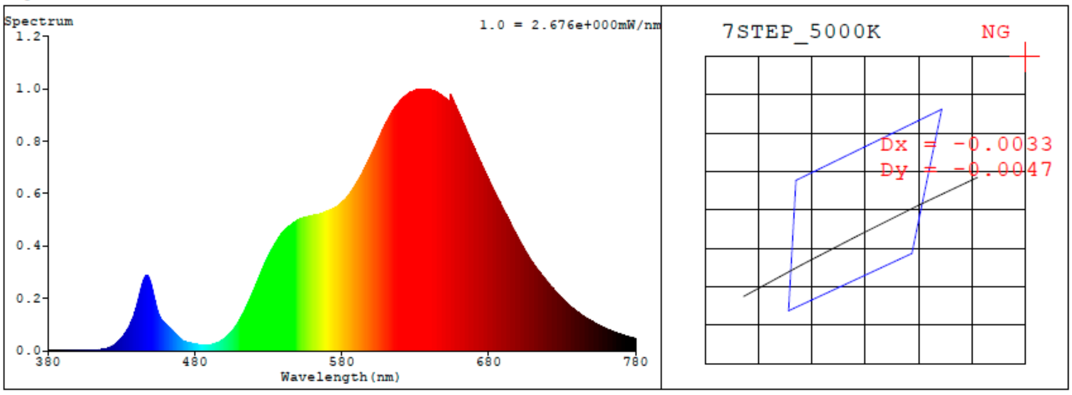

Colorimetric Parameters

Chromaticity Coordinate: x = 0.5017 y = 0.4105 / u' = 0.2899 v' = 0.5337 (duv=-1.52e-03)   Dx,Dy:-0.0033,-0.0047

CCT= 2207K   Prcp WL: Ld=587.7nm   Purity=73.8%

Peak WL: Lp=636nm FWHM: =143.1nm Ratio:R=31.4% G=67.9% B=0.7%

Render Index: Ra = 87.0 AvgR = 82.7

EEI: -

R1 =91	R2 =90	R3 =81	R4 =88	R5 =86	R6 =82	R7 =92	
R8 =85	R9 =65	R10=70	R11=83	R12=58	R13=90	R14=87	R15=90

LEVEL:---   WHITE:OUT

Photometric & Radiometric Parameters

Flux = 99.013 lm Fe = 393.80 mW

Supplementary Figure S1. Photometer assessment results

Supplementary Table S1. Sequence of primers used for real-time reverse transcription polymerase chain reaction.

Genes	Accession No	Primer sequence	Condition
CLOCK	NM_004898.3	F: TCTTGACCTTATGCCATTCCA<br>R: CTTATGCTTTGTTGCTGTCAACC	60°C
BMAL1	NM_001178.4	F: GTAACCTCAGCTGCCTCGTC<br>R: TAGCTGTTGCCCTCTGGTCT	60°C
PER1	NM_002616.2	F: ACAAGCAAATACTTTGGCAGCATC<br>R: CCTGCTTCAGCACAGAGGTCA	60°C
PER2	NM_022817.2	F: CGTTGGAACCACCCAGACATC<br>R: ATGCAGTCGCAAGCTGTCAGA	60°C
PER3	NM_001289861.1	F: GCGCACTCATATATGCTAAGCCTTC<br>R: CTGCGACAAGCACCAGTTTCA	60°C
CRY1	NM_004075	F: TCTGGCATCAGTACCTTCTAATCC<br>R: CTGTGTGTCCTCTTCCTGACTAG	60°C
CRY2	NM_001127457.2	F: GACCAGGTTGCAGTGGCGTA 60°C<br>R: GCCCTGGAAGCCAACAGAATAA	

Supplementary Table S2. Changes in sleep parameters and mood in the two groups before and after the two-week period

Variable	Light therapy group (n = 20)				Control group (n = 20)				Difference in difference (95% CI)†	p-value†
	Pre	Post	Diff	p-value*	Pre	Post	Diff	p-value*		
MEQ	47.25 ± 7.83	48.65 ± 8.46	1.40 ± 4.39	0.170	42.70 ± 7.16	42.95 ± 7.90	0.25 ± 4.25	0.795	1.49 (-1.70, 4.69)	0.350
ESS	7.10 ± 3.02	6.30 ± 3.23	-0.80 ± 2.28	0.134	6.80 ± 3.72	5.95 ± 3.43	-0.85 ± 2.11	0.087	0.01 (-1.46, 1.49)	0.984
ISI	10.25 ± 5.28	6.60 ± 4.28	-3.65 ± 4.18	**0.001**	8.20 ± 4.50	5.70 ± 3.10	-2.50 ± 2.82	0.001	-0.60 (-2.51, 1.32)	0.531
PHQ	6.60 ± 4.33	4.80 ± 4.19	-1.80 ± 2.98	**0.014**	5.15 ± 6.18	4.05 ± 3.98	-1.10 ± 3.26	0.148	-0.05 (-1.75, 1.66)	0.956
PSQI	10.80 ± 3.86	7.60 ± 2.93	-3.20 ± 3.76	**0.001**	8.95 ± 2.82	8.35 ± 2.18	-0.60 ± 2.46	0.289	-2.00 (-3.58, -0.43)	**0.014**
SSS	2.55 ± 0.76	2.50 ± 1.19	-0.05 ± 1.19	0.853	2.30 ± 0.86	2.15 ± 0.59	-0.15 ± 0.81	0.419	0.16 (-0.51, 0.82)	0.634

Abbreviations; CI, Confidence interval; MEQ, Morningness-Eveningness Questionnaire; ESS, Epworth Sleepiness Scale; ISI, Insomnia Severity Index; PHQ, Patient Health Questionnaire; PSQI, Pittsburgh Sleep Quality Index; SSS, Stanford Sleepiness Scale

*p-values calculated using paired t-test.

†Difference in difference and p-value calculated using linear regression to adjust for the baseline value and body mass index.
